# Production of a polyclonal antibody against inosine-uridine preferring nucleoside hydrolase of *Acanthamoeba castellanii* and its access to diagnosis of *Acanthamoeba* keratitis

**DOI:** 10.1371/journal.pone.0239867

**Published:** 2020-09-30

**Authors:** So-Min Park, Hae-Ahm Lee, Ki-Back Chu, Fu-Shi Quan, Su-Jung Kim, Eun-Kyung Moon

**Affiliations:** 1 Department of Biomedical Science, Graduate School, Kyung Hee University, Seoul, Korea; 2 Medical Research Center for Bioreaction to Reactive Oxygen Species and Biomedical Science Institute, School of Medicine, Graduate School, Kyung Hee University, Seoul, Korea; 3 Department of Medical Zoology, Kyung Hee University School of Medicine, Seoul, Korea; 4 Department of Biomedical Laboratory Science, Daegu Health College, Daegu, Korea; Instituto Butantan, BRAZIL

## Abstract

*Acanthamoeba* keratitis (AK) is a rare disease but its prevalence throughout the globe continues to grow, primarily due to increased contact lens usage. Since early-stage symptoms associated with AK closely resemble those from other corneal infections, accurate diagnosis is difficult and this often results in delayed treatment and exacerbation of the disease, which can lead to permanent visual impairment. Accordingly, developing a rapid *Acanthamoeba*–specific diagnostic method is highly desired. In the present study, a rapid and differential method for AK diagnosis was developed using the secretory proteins derived from the pathogenic *Acanthamoeba*. Among the vast quantities of proteins secreted by the pathogenic *Acanthamoeba*, an open reading frame of the inosine-uridine preferring nucleoside hydrolase (IPNH) gene was obtained. After expressing and purifying the IPNH protein using the pGEX 4T-3 vector system, mice were immunized with the purified proteins for polyclonal antibody generation. Western blot was performed using protein lysates of the human corneal cell, non-pathogenic amoeba, pathogenic amoeba, and clinical amoeba isolate along with lysates from other causes of keratitis such as *Staphylococcus aureus*, *Pseudomonas aeruginosa*, and *Fusarium solani* to confirm *Acanthamoeba*-specificity. Western blot using the polyclonal IPNH antibody revealed that IPNH was *Acanthamoeba*-specific since these proteins were only observed in lysates of *Acanthamoeba* origin or its culture media. Our findings indicate that the IPNH antibody of *Acanthamoeba* may serve as a potential agent for rapid and differential AK diagnosis.

## Introduction

*Acanthamoeba* keratitis (AK) is a rare but painful and sight-threatening ocular infection, which frequently occur in contact lens wearers [1, 2]. Currently, it is estimated that approximately 2 million contact lens wearers in the United States are susceptible to AK [3] and outbreaks are continuously reported in multiple developed countries [4–6]. The incidence rates of AK have been steadily increasing over the decades and accordingly, the need for proper diagnosis and treatment cannot be overstated enough. Nevertheless, diagnosis remains difficult as AK is frequently misdiagnosed as keratitis of viral, bacterial, or fungal origin, which stems from the fact that the clinical manifestations of these origins are strikingly similar to that of AK [7]. Consequently, this inaccurate diagnosis leads to treatment failure and prolonged infection, which can lead to permanent visual impairment in patients. To this extent, developing a diagnostic method that accurately distinguishes between AK and keratitis caused by other microbial pathogens would have a profound impact.

Current diagnostic techniques for AK include *in vivo* confocal microscopy, polymerase chain reaction (PCR) amplification, histopathological examinations, and *Acanthamoeba* culture on agar plate [7, 8]. Confocal microscopy is a sensitive and rapid diagnostic method that can easily detect the distinct double-wall feature of the *Acanthamoeba* cysts, albeit with several drawbacks such as difficulties associated with distinguishing trophozoites from leukocyte or keratinocyte nuclei [9, 10]. PCR or real-time PCR amplification is another sensitive and rapid diagnostic method but even this method can be misleading as it cannot distinguish between nucleic acid from living or dead *Acanthamoeba* cells, the latter of which often leads to the acquisition of false-positive results [11, 12]. The presence of *Acanthamoeba* could be verified by histopathological analysis or multiple staining procedures including calcofluor-white stain and Giemsa stain, but these invasive methods require corneal scrapping or explanted tissue [8, 13]. Culture-based detection remains the gold-standard diagnosis for AK, where corneal scrapings from patients suspected with *Acanthamoeba* infection are grown on *Escherichia coli*-enriched agar plates.

Diagnostic methods for various ocular diseases, which involve antibody testing have been reported with remarkably high sensitivity. For example, combining PCR and *Toxoplasma gondii*-specific antibodies to diagnose ocular toxoplasmosis was reported with a high rate of sensitivity and specificity in several investigations [14]. Monoclonal antibody-based testing kit for chlamydia conjunctivitis in infants was also documented to be highly sensitive and specific [15]. Another monoclonal antibody-based immunoassay enabled rapid detection of ocular herpes simplex virus infection [16]. As antibody-based diagnostics have advanced, the development of an antibody-bsaed protein microarray is being attempted [17]. Antibody-based proteomics and biomarker research using body fluids and tissues provide improved assay specificity and sensitivity [18].

Yet, only a limited number of studies have investigated antibody-based AK diagnosis to date. Earlier studies have reported the importance of anti-*Acanthamoeba* IgA and its potential role for differentiating between pathogenic and non-pathogenic types [19, 20]. *Acanthamoeba*-specific monoclonal antibodies successfully reacted with the cysts of virulent *Acanthamoeba* strains, signifying its potential for the diagnosis of AK [21]. The monoclonal antibody generated against a mannose-binding protein (MBP) of *A*. *culbertsoni* could also be used for identifying the presence of *Acanthamoeba* in clinical isolates [22]. Although these studies elucidated the potential of antibody-based testing for AK diagnosis, one limitation of the aforementioned works is that the authors did not address the *Acanthamoeba*-specificity of their antibodies, which is crucial for discerning keratitis of bacterial, viral, or fungal origins from that incurred by *Acanthamoeba*. Therefore, developing an antibody-based assay that specifically recognizes keratitis of *Acanthamoeba* origin would further contribute to AK diagnosis.

In the previous study, expression levels of secretory proteins were compared between non-pathogenic and pathogenic strains of *A*. *castellanii*, and pathogenic strain revealed 34 increased proteins and 7 qualitatively increased proteins [23]. Among 34 increased proteins of pathogenic *Acanthamoeba*, inosine-uridine preferring nucleoside hydrolase (IPNH) was selected for further work. In this study, IPNH was isolated from an expression system in *E*. *coli*, and polyclonal antibody against this protein was generated in mice. *Acanthamoeba*-specificity of this antibody was confirmed by assessing its interaction with antigens from various species, including human corneal epithelial cells, non-pathogenic *Acanthamoeba*, pathogenic *Acanthamoeba*, clinical isolate of *Acanthamoeba* and other causes of keratitis such as *Staphylococcus aureus*, *Pseudomonas aeruginosa*, and *Fusarium solani*. Findings of this study reveal the presence of *Acanthamoeba*-specific protein, which could aid AK diagnosis in clinical settings.

## Materials and methods

### Cell culture

Non-pathogenic and pathogenic strains of *Acanthamoeba castellanii* Castellani were obtained from the American Type Culture Collection (ATCC30011 and ATCC 30868) [24–26], and the clinical isolate was acquired from a patient diagnosed with AK at the Kyung Hee University Hospital (Seoul, Republic of Korea). *Acanthamoeba* trophozoites were cultured axenically in PYG medium (20 g proteose peptone, 1 g yeast extract, 0.1M glucose, 4 mM MgSO_4_, 0.4 mM CaCl_2_, 3.4 mM sodium citrate, 0.05 mM Fe(NH_4_)_2_(SO_4_)_2_, 2.5 mM Na_2_HPO_4_, and 2.5 mM K_2_HPO_4_) at 25°C. The culture media was harvested after cultivation for 7 days. Human corneal epithelial (HCE) cell (American Type Culture Collection PCS-700–010) was cultured in endothelial cell growth medium kits (KGM BulletKit; Lonza, Portsmouth, NH) at 37℃ with 5% CO_2_. *Pseudomonas aeruginosa* (NCCP 16091), *Staphylococcus aureus* (NCCP 15920), and *Fusarium solani* (NCCP 32678) were obtained from the Korea Centers for Disease Control & Prevention. *P*. *aeruginosa* and *S*. *aureus* were streaked on Brain Heart Infusion (BHI) agar plates and incubated at 37℃ for 24 h. *F*. *solani* was streaked on Sabouraud Dextrose (SD) agar plates and incubated at 25℃ for 5 days.

### Cloning, expression, and purification of IPNH

Primers were designed using the full-length gene sequence of *A*. *castellanii* Neff inosineuridine preferring nucleotide hydrolase (IPNH) provided by the NCBI database (GenBank accession no: XP_004334078). The coding sequence of *A*. *castellanii* (ATCC 30868) IPNH was amplified by polymerase chain reaction using the following primers: 5’-ATGACCGCGAGCAAGTCGTT-3’ (forward primer), and 5’-CTACCAGCGGCGTCGCCTGT-3’ (reverse primer). *Escherichia coli* BL21 (DE3) was used as an expression host of pGEX 4T-3 GST expression vector (GE Healthcare, Buckinghamshire, UK). Isopropyl-β-D-thiogalactopyranoside (IPTG) was added to a final concentration of 1 mM for expression of GST-IPNH fusion protein in *E*. *coli*, and incubated for 4 h. Protein expressions were confirmed using sodium dodecyl sulfate polyacrylamide gel electrophoresis (SDS-PAGE) and Coomasie blue staining (Elpisbio, Daejeon, Korea). Target protein was extracted using the EzWay™ PAG Protein Elution Kit V_2_ (KOMABIOTECH, Seoul, Korea). Protein samples were concentrated by Amicon Ultra-4 centrifugal filter device (Merck Millipore, Burlington, MA, USA).

### Immunization of the IPNH to BALB/c mice

Six-week-old male BALB/c mice (n = 2) were purchased from Koatech (Pyeongraek, Korea) and mice were injected subcutaneously with 50 μg of antigen mixed with 50 μl of Freund’s complete/incomplete adjuvant (Sigma-Aldrich, St. Louis, MO, USA) at three week intervals. Three weeks after 3^rd^ immunization, mice were anesthetized and cardiac puncture was performed for serum collection. All animal procedures performed in this study were reviewed, approved, and supervised by Kyung Hee University Institutional Animal Care and Use Committee (permit number: KHSASP-19-035).

### Determining the *Acanthamoeba* specificity of the IPNH antibody

*Acanthamoeba*-specificity of the antibody was determined by western blotting. Briefly, culture media of *Acanthamoeba*, and whole cell lysates of HCE cells, *Acanthamoeba* spp., *P*. *aeruginosa*, *S*. *aureus*, and *F*. *solani* were prepared using the Pro-Prep protein lysis buffer (iNtRON BioTechnology, Seongnam, Korea). Protein concentrations were determined using the Bradford reagent (BIO-RAD, CA, USA). Proteins were resolved by SDS-PAGE and transferred on to a nitrocellulose (NC) membrane. The membrane was blocked with 5% skim milk in TBST (25 mmol/L Tris base, 150 mmol/L NaCl, and 0.1% Tween 20) buffer for 1 h at RT, and incubated overnight at 4 ℃ with the IPNH polyclonal antibody. On the next day, the membrane was washed for 5 minutes with TBST three times and incubated with horseradish peroxidase (HRP)-conjugated anti-mouse antibody (Sigma-Aldrich, St. Louis, MO, USA) at room temperature for 1 h. Membranes were washed and after exposure to enhanced chemiluminescence (ECL; Thermo Fisher, MA, USA), protein expressions were detected on an x-ray film in the darkroom.

## Results

### Identification of IPNH of *A*. *castellanii*

**Fig 1:**
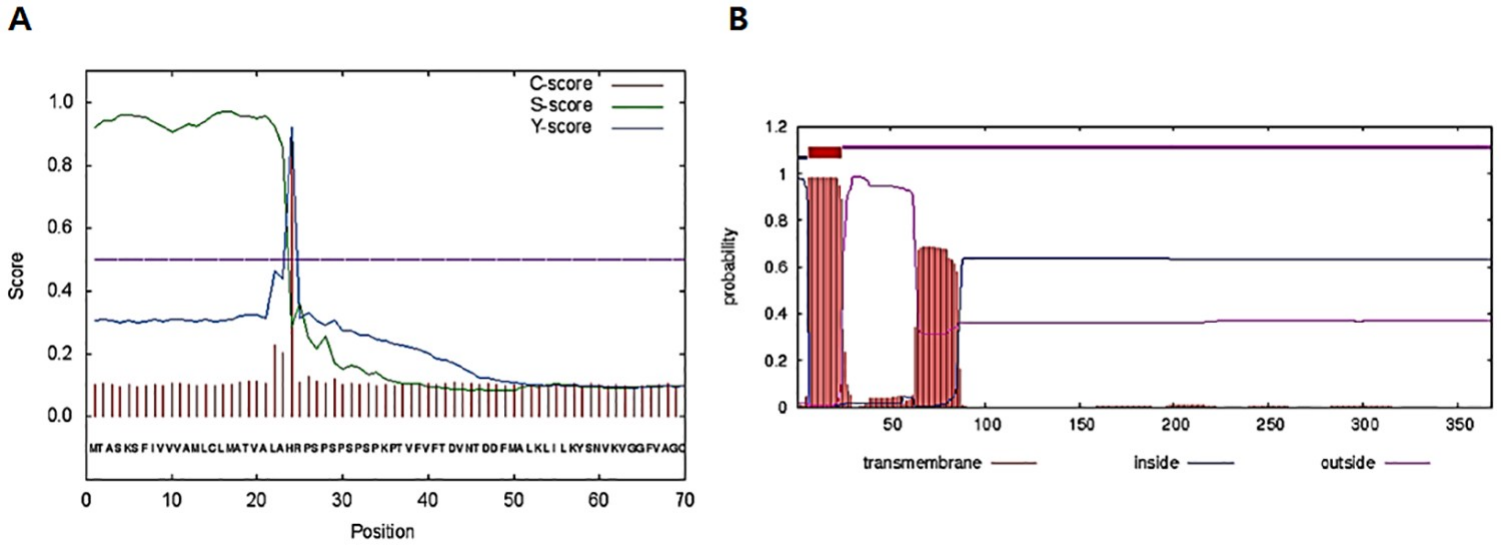
Peptide analysis of IPNH. Analysis of the deduced amino acid sequence of IPNH showed the existence of signal peptide (A) and transmembrane domain (B).

In our previous investigation, secretory proteins of *A*. *castellanii* non-pathogenic strain and pathogenic strain were compared by 2-dimensional polyacrylamide gel electrophoresis [23]. In that study, one protein among 41 up-regulated proteins in the pathogenic strain was selected (S1 Fig). The full-length open reading frame of that gene consists of 1,107 bp and encodes 368 amino acids with a calculated mass of 40.48 kDa (GenBank accession number MN630521). Protein homology search results revealed that this protein showed 92% similarity with inosineuridine preferring nucleoside hydrolase (IPNH) family protein from *A*. *castellanii* Neff (GenBank accession number XP_004334078.1). Peptide analysis was performed using the SignalP 4.1 server database, which predicted the signal peptide and the transmembrane domain to be located near the N-terminus at amino acid positions 1 to 23 and 7 to 24, respectively (Fig 1A and 1B).

### Production of polyclonal antibodies

**Fig 2:**
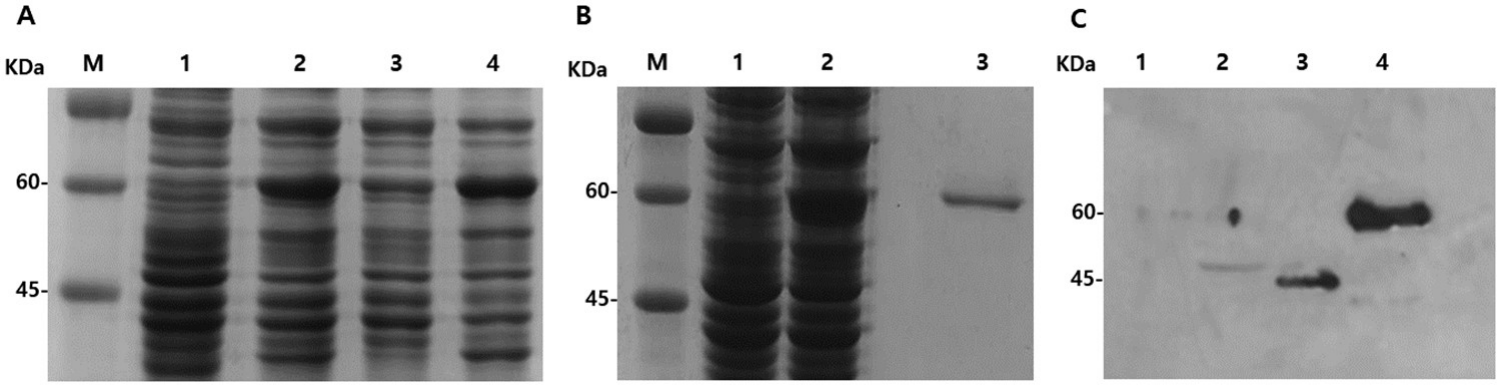
Production of polyclonal antibody against IPNH. Protein expression was confirmed by SDS-PAGE (A). M: protein size marker, lane 1: uninduced total protein, lane 2: induced total protein, lane 3: induced soluble protein, and lane 4: induced insoluble protein. The expressed GST tagged protein (67.1 kDa) was purified (B). Lane 1: uninduced total protein, lane 2: induced total protein, and lane 3: purified target protein. Western blot was performed to confirm the antibody generation (C). Lane 1: HCE cells (30 μg), lane 2: cell lysate of *A*. *castellanii* (30 μg), lane 3: cultured media of *A*. *castellanii* (2 μg), and lane 4: purified target protein (2 μg).

To assess the potential application of IPNH protein for *Acanthamoeba* keratitis diagnosis, we produced a polyclonal antibody against IPNH of *A*. *castellanii* using pGEX 4T-3 expression vector system. The full-length GST-tagged proteins (67.1 kDa) were successfully expressed with 1 mM IPTG (Fig 2A). Very few GST-tagged proteins were induced from soluble bodies (lane 3), and most of the target proteins appeared at the insoluble fraction (lane 4). Insoluble GST-IPNH fusion protein was purified by gel extraction, and elution fraction was analyzed by SDS-PAGE (Fig 2B). Lane 3 showed the presence of eluted GST-IPNH proteins. Mice were immunized thrice for IPNH antibody generation and the resulting antibody reactivity was evaluated through western blot using human corneal epithelial (HCE) cell and *Acanthamoeba* (Fig 2C). Western blot results demonstrated that the polyclonal IPNH antibody did not react with HCE cell lysates (lane 1) but reacted with *A*. *castellanii* cell lysate (lane 2) and cultured media of *A*. *castellanii* (lane 3). Eluted GST-IPNH proteins were loaded as a positive control (lane 4). The estimated size of the GST-IPNH fusion protein was 67.1 kDa (lane 4), and the full-length amino acids of IPNH was calculated to be 48.48 kDa (lane 2) whereas the secretory IPNH in culture media was estimated to be 37.95 kDa (lane 3).

### Specificity of a polyclonal IPNH antibody

**Fig 3:**
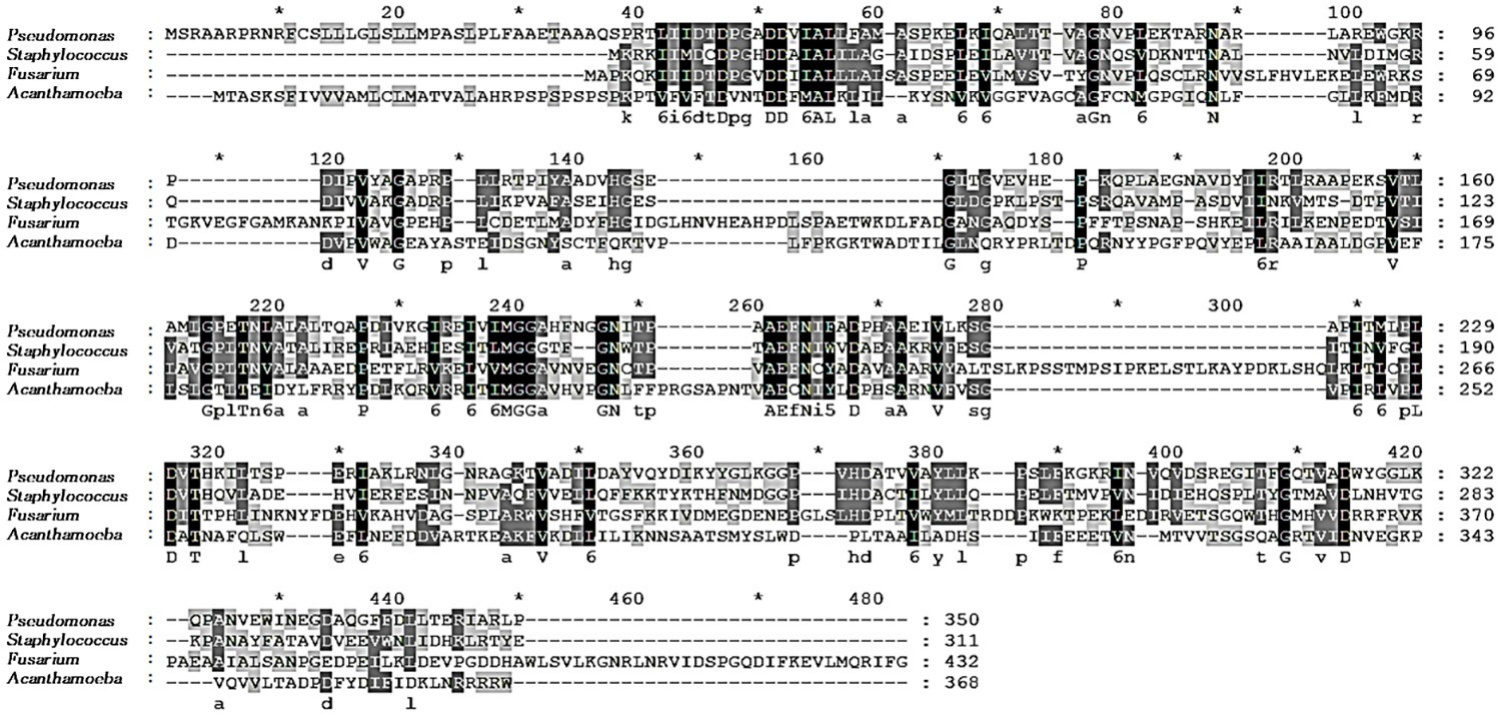
Alignment of the IPNH amino acid. Aligned amino acid sequences of *P*. *aeruginosa* (WP_023095033.1), *S*. *aureus* (LN626917.1), *F*. *avenaceum* (KIL94995.1), and *A*. *castellanii* (MN630521) were compared. ClustalX multiple sequence alignment was used to produce the alignment, and the degree of conservation is represented by different shading.

**Fig 4:**
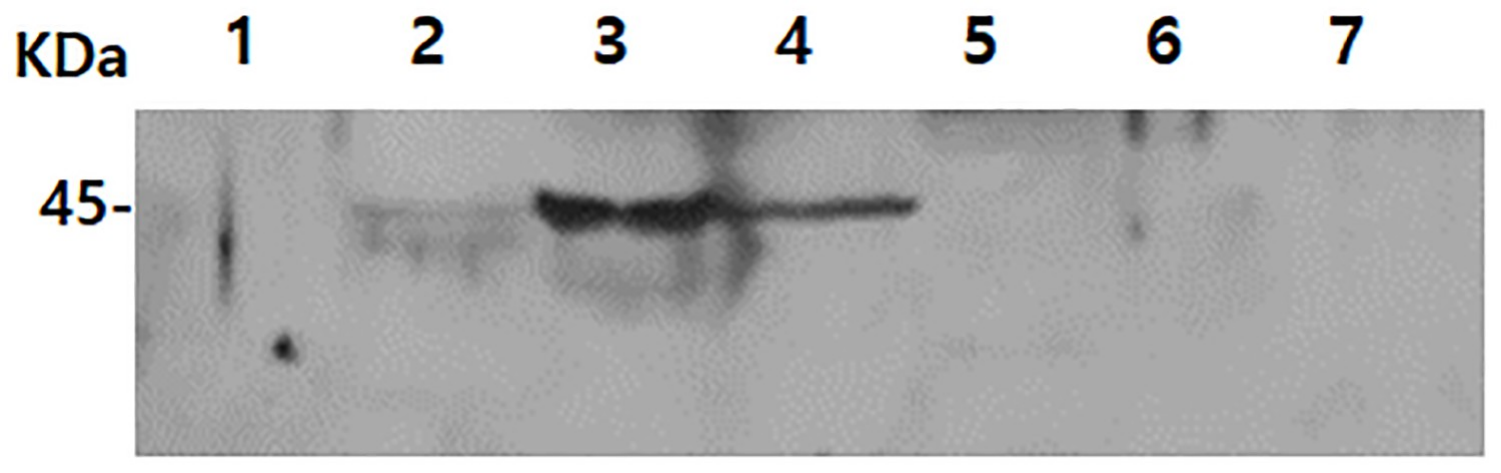
Specific antibody response of IPNH. The differential diagnosis of *Acanthamoeba* keratitis was confirmed by western blot using a polyclonal antibody against IPNH of *A*. *castellanii*. Lane 1: HCE cells (30 μg), lane 2: non-pathogenic *A*. *castellanii* (30 μg), lane 3: pathogenic *A*. *castellanii* (30 μg), lane 4: clinical isolate of *Acanthamoeba* (30 μg), lane 5: *P*. *aeruginosa* (30 μg), lane 6: *S*. *aureus* (30 μg), and lane 7: *F*. *solani* (30 μg).

To determine whether the polyclonal IPNH antibody was capable of differentiating between other causes of keratitis or not, amino acid sequence comparison and western blot using protein lysates of *S*. *aureus*, *P*. *aeruginosa*, and *Fsusarium* spp. were performed. Amino acid sequences comparison results confirmed that extremely low similarities were shared between the IPNH sequences of *A*. *castellanii* and those of *S*. *aureus*, *P*. *aeruginosa*, and *F*. *avenaceum* (Fig 3). Because the IPNH gene sequence from *F*. *solani* was unreported in the GenBank database, *F*. *avenaceum* gene sequence was used in its place. Based on homology search results, the amino acid sequences of IPNH of *A*. *castellanii* shared 22.7%, 18.5%, and 16.6% sequence similarities with IPNH of *P*. *aeruginosa*, *S*. *aureus*, and *F*. *avenaceum*, each respectively. Western blot results further confirmed the *Acanthamoeba*-specificity of polyclonal IPNH antibody, which did not interact with proteins of other organisms (Fig 4). IPNH was successfully detected from *Acanthamoeba* cell lysates but not from HCE cells, *P*. *aeruginosa*, *S*. *aureus*, and *F*. *solani*. A faint band was observed in the non-pathogenic *Acanthamoeba* (lane 2), and strong bands were observed in pathogenic *Acanthamoeba* (lane 3) and the clinical isolate of *Acanthamoeba* (lane 4).

## Discussion

Approximately 23% of the patients diagnosed with AK suffer concomitant co-infection with a virus, bacteria, or fungi, and 75–90% of all patients are misdiagnosed since clinical symptoms associated with AK during the early stages of the disease are strikingly similar to keratitis caused by other pathogens [7]. Given this nature of the disease, a highly specific diagnostic method for AK is in dire need. Current diagnostic techniques for *Acanthamoeba* keratitis (AK) such as PCR, immunohistochemistry, and culture require biopsies or corneal scrapes [21]. Accurate diagnosis of AK needs deep corneal scraping and biopsy and this procedure, paired with repeated sampling, incurs excruciating pain in patients. Common symptoms of AK patients include massive pain, photophobia, and lacrimation [27]. Based on the fact that AK patients undergo extensive tear production, we focused on raising an antibody against a secretory protein which could be detected from the patients’ tears for ease of diagnosis. Among the numerous proteins secreted by the pathogenic *Acanthamoeba* is inosine-uridine preferring nucleoside hydrolase (IPNH), a protein that remains uncharacterized to date. In the current study, the potential application of IPNH for AK diagnosis was investigated by generating polyclonal antibodies against it in mice.

IPNH of *A*. *castellanii* has signal peptides and transmembrane domain (Fig 1), which signify that the protein may be easily secreted from the trophozoites of *Acanthamoeba*. In support of this, the pathogenic strain of *A*. *castellanii* has been found to secrete a large amount of IPNH proteins [23]. From this finding, we hypothesized that measurable quantities of IPNH proteins are discharged through the tears of AK patients which implies that the IPNH antigen present in tears can be used for AK diagnosis. Furthermore, since corneal scraping will not be required, this method will also incur less physical discomfort in patients during the sampling process.

Cloning of the IPNH gene in *A*. *castellanii* revealed that IPNH is composed of 1,107 nucleotides that encode 368 amino acids. The amino acid sequence homology between *A*. *castellanii* and other species that cause corneal inflammation was very low. Evidently, the polyclonal antibodies generated against IPNH reacted with *A*. *castellanii* lysate and cultured media, but these antigen-antibody interactions were not observed from the lysates of HCE cells, *P*. *aeruginosa*, *S*. *aureus*, and *F*. *solani* as we initially anticipated (Fig 4). One possible explanation for this arises from the biochemical nature of protozoan metabolism. Because *de novo* purine biosynthesis is non-existent in protozoan parasites such as *Trypanosoma*, these organisms rely on nucleoside hydrolases for purine salvaging from hosts [28, 29]. However, genes encoding nucleoside hydrolase as observed in protozoans have not been reported from higher eukaryotes, especially mammals [30]. Additionally, the number of reports documenting inosine-uridine nucleoside hydrolase expressions occurring in bacteria seems to be extremely limited. Currently, these enzymes have only been detected from *E*. *coli*, *Bacillus anthracis*, *Salmonella enterica* serovar Typhimurium [31–34]. Based on these earlier findings, the IPNH antibody used in the current investigation is considered to be highly selective and specific for the protein of *Acanthamoeba* origin.

The diagnostic method using the IPNH antibody could be rapid and accurate, but there are a few limitations of the current study that need to be addressed. Although the IPNH antibody used in the current study can be a differential diagnostic method of AK as the antibody specifically interacted with the IPNH of *Acanthamoeba*, it does not account for cross-species interaction amongst the genus *Acanthamoeba*. To date, several species of *Acanthamoeba* have been identified from ocular infection, which includes *A*. *castellanii*, *A*. *polyphaga*, *A*. *rhysodes*, *A*. *culbertsoni*, *A*. *hatchetti*, *A*. *griffini*, *A*. *quina* and *A*. *lugdunensis* [35]. Of the 8 species listed above, *A*. *castellanii* and *A*. *polyphaga* are the species most commonly associated with *Acanthamoeba* keratitis [36]. The IPNH generated in the present study did not investigate the potential cross-reactivity between similar species, as this was beyond the scope of this investigation. Another issue associated with the study is the dilution of secretory IPNH by the patients’ tears. Highly diluted protein samples can limit the detection capabilities of the IPNH antibody, which can lead to false-negative results. Therefore, it seems necessary to consider the minimum detectable concentration of IPNH, and the combination of IPNH antibody with anti-*Acanthamoeba* IgA or other *Acanthamoeba*-specific antibodies against secretory proteins. Overall, our results suggest that the polyclonal antibody against the secretory IPNH of *A*. *castellanii* may be useful for the differential diagnosis of AK. Further studies that address the aforementioned limitations should be performed to enable its clinical application.

## Supporting information

S1 Fig 2DE analysis of secretory proteins in *A*. *castellanii*. Secretory proteins between non-pathogenic strain (A) and pathogenic strain (B) of *Acanthamoeba* were compared. Among the highly expressed proteins in the pathogenic *Acanthamoeba*, one protein spot marked (*) in 2DE gel was selected for further analysis which was identified as IPNH.
(TIF)

S1 Raw image (PDF)

S2 Raw image (PDF)

S3 Raw image (PDF)

S4 Raw image (PDF)
