# Supplementary figures and images for: Production of a polyclonal antibody against inosine-uridine preferring nucleoside hydrolase of Acanthamoeba castellanii and its access to diagnosis of Acanthamoeba keratitis
Source: PLoS One. 2020 Sep 30;15(9):e0239867. doi: 10.1371/journal.pone.0239867 (PMC7526901; doi:10.1371/journal.pone.0239867)

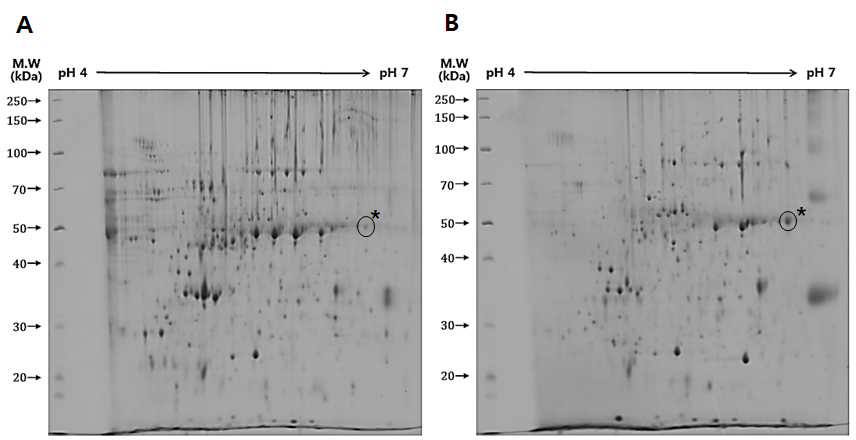

Supplement: S1 Fig — Secretory proteins between non-pathogenic strain (A) and pathogenic strain (B) of Acanthamoeba were compared. Among the highly expressed proteins in the pathogenic Acanthamoeba, one protein spot marked (*) in 2DE gel was selected for further analysis which was identified as IPNH. (TIF) [file pone.0239867.s001.tif]

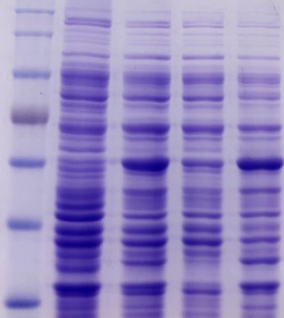

Supplement: S1 Raw image — (PDF) [file pone.0239867.s002.pdf]

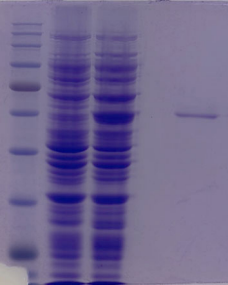

Supplement: S2 Raw image — (PDF) [file pone.0239867.s003.pdf]

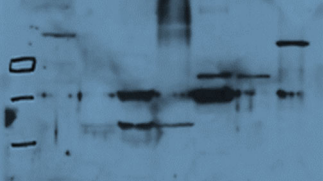

Supplement: S4 Raw image — (PDF) [file pone.0239867.s005.pdf]
