# Supplementary material for: Production of a polyclonal antibody against inosine-uridine preferring nucleoside hydrolase of Acanthamoeba castellanii and its access to diagnosis of Acanthamoeba keratitis
Source: PLoS One. 2020 Sep 30;15(9):e0239867. doi: 10.1371/journal.pone.0239867 (PMC7526901; doi:10.1371/journal.pone.0239867)
Supplement: S3 Raw image — (PDF) [file pone.0239867.s004.pdf]

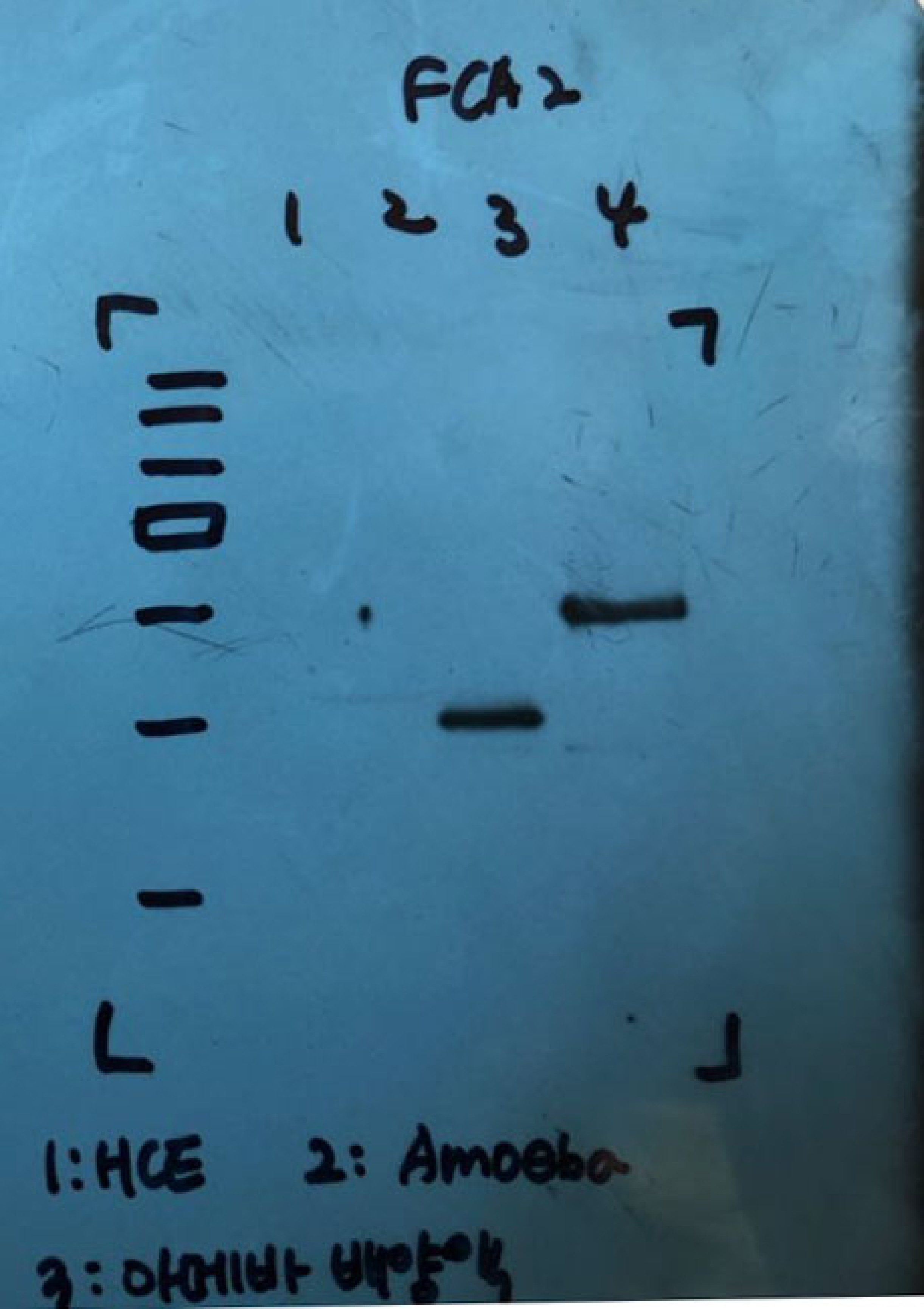
FCA2
1 2 3 4
1:HCE 2: Amoeba
3: 아메바 배양액
